# Thinking and Enacting the Patient Medical Home Under Pandemic Conditions: A Qualitative Study From Primary Care in Alberta, Canada

**DOI:** 10.1177/21501319241236007

**Published:** 2024-04-16

**Authors:** Myles Leslie, Brian Hansen, Rida Abboud, Caroline Claussen, Fariba Aghajafari

**Affiliations:** 1 University of Calgary, Calgary, AB, Canada; 2 Alberta Health Services, Calgary, AB, Canada; 3 Co-RIG Project Consultant, Calgary, AB, Canada

**Keywords:** patient medical home, primary care, primary health care, COVID-19, qualitative

## Abstract

**Background:**

The COVID-19 (C19) pandemic shocked primary care systems around the world. Those systems responded by supporting patients in the community, and acute care facilities in crisis. In Canada, the Patient Medical Home (PMH) is a widely adopted care model that aims to operationalize the tenets and principles of Primary Health Care (PHC) as developed since the Alma-Ata Declaration. This paper describes how personnel working in and with Primary Care Networks (PCNs) in Alberta, Canada deployed the PMH model and its underlying PHC principles to frame and respond to the C19 shock.

**Methods:**

Using purposive and snowball sampling techniques, we interviewed 57 participants who worked in public health and primary care, including community-based family physicians. We used interpretive description to analyze the interviews.

**Results:**

PCN staff and physicians described how the PMH model was foundational to normal operations, and how C19 responses were framed by the patient-centric, team-delivered, and continuous care principles the model shares with PHC. Specifically, participants described ensuring access to care, addressing the social determinants of health, being patient centered, and redeploying and expanding PHC teams to accomplish these goals.

**Discussion:**

Delivering PHC through the PMH allowed physicians and allied health staff to deliver patient-centered, team-based, holistic bio-medical services to Albertans. In tailoring services to meet the specific social and health needs of the populations served by each PCN, healthcare providers were able to ensure relevant support remained available and accessible.

## Background

Primary care teams have played significant roles in the response to COVID-19 (C19), ranging from the in-community management of patients^1,2^ to vaccine counseling and delivery.^3-7^ Striving to deliver Primary Health Care (PHC) broadly and holistically conceived,^8,9^ those teams have protected the capacity of acute care facilities^10^ and integrated with other elements of the health system helping to achieve resilience.^11-14^ The shock of the pandemic to PHC provision has been unique in its sustained severity,^15,16^ highlighting and exacerbating longstanding challenges to the provision of PHC including burnout amongst existing clinicians^17-19^; threats to the financial viability of practices^20,21^; and a decreased flow of new physicians into the practice of family medicine.^22-24^ In this context of new and old challenges, the pandemic’s effects on the delivery of PHC-focused care models are not well understood. This paper takes Canada’s Patient Medical Home (PMH)^25^ care model as a particular example of how the tenets and principles of PHC were shaped by, and put into action during, the C19 shock. It highlights how the PMH was not just a rhetorical trope for those providing PHC, but a way of imagining and organizing their response to the pandemic. We show how the PMH underpinned more effective integration of primary and acute care operations and draw out lessons for jurisdictions beyond where the study took place.

This paper analyzes qualitative data to describe how the PMH became a shared way of thinking about and responding to the pandemic amongst PHC stakeholders in Alberta, Canada. We begin by describing the primary care policy environment in Alberta, and then move to a brief history of the PMH, tracing out its focus on *patient centrism, team-based medicine*, and a *continuous home* or point of contact for patients. We next show how these concepts were enacted by stakeholders who took a shared rhetoric and turned it into action. Illustrating how PHC principles and PMH policies advanced under pandemic conditions, we pose questions for those designing reform and transformation policy for a post C19 environment. Following how the tenets of PHC were put into action through the PMH during the pandemic reveals important considerations for those working to transform primary care through policy.

### Primary Health Care in Alberta

The delivery of PHC in Alberta is a provincial policy goal,^26^ supported by dedicated structures in the health system. Alberta is the only province in Canada to operate a single central health authority—Alberta Health Services (AHS). AHS’ dominant focus is on running acute care and long-term care facilities, as well as the provincial ambulance and public health services, but it does staff a dedicated PHC unit that aims to co-ordinate and integrate primary care into these broader operations. Recent moves toward reform appear set to diminish AHS’ role and empower a freestanding primary care organization^27-29^ but these have not yet taken concrete shape. If this is the central system’s support for PHC, then there are also community-based structures—the Primary Care Networks (PCNs). The PCNs act as bridges between AHS and the independent-contractor physicians who provide the vast majority of primary care to Albertans, and who bill the provincial government on a fee-for-service (FFS) basis.^30^

The province’s more than 3800 independent primary care physicians have the option to join 1 of 40 PCNs. The majority opt-in, with their choice triggering a capitation payment based on the size of their patient panel, which is then made by the ministry of health to the selected PCN. While physicians are still reimbursed for their services through FFS, or in rare cases, other alternative methods of payment, the capitation funding is routed toward the PCNs In this way both FFS and capitation models run concurrently, with the PCNs being held accountable for the funds they receive on behalf of their member physicians through a range of requirements that include the delivery of the PMH. Their a particular focus as they operationalize the PMH, and their progress is measured, is on attaching patients and so improving Albertans’ access to PHC.^26,31-33^ Initially focused on improving access and the patient-centeredness of care for patients with complex chronic conditions, the PCNs have added co-planning service delivery with local AHS authorities to meet the particular needs of the populations in their geographic catchments.

As much as the PCNs have evolved to be, if not the glue, then at least an interaction point between the acute care-focused AHS system and primary care, barriers to achieving integrated care coordination, and so delivering PHC, have persisted. Those barriers include an administrative focus on acute care and divergent funding and accountability models. As in the rest of Canada, Alberta focuses its time and money on the oversight and delivery of acute care. This specialization in, and emphasis on, building hospitals and managing in-patient services can be traced to the original negotiations and social contract that gave rise to the nation’s various publicly funded health systems.^34^ At the time that Canada’s modern health systems were established, primary care physicians (PCPs) preferred the status of an independent contractor working on a FFS basis, and this set them apart from the employee status and salaries of hospital-based physicians. Beyond paying more attention to paid employees working in acute care than to independent contractors working in longitudinal care, the 2 funding models have been deployed with quite different accountability expectations. Despite calls to draw primary and acute care into closer integration^35^ bridging those divergent expectations for performance has proved to be challenging.^36,37^

If the PCNs—striving to support their physician members in the delivery of the PMH—were the primary contact point between the isolated primary and acute care elements of Alberta’s system prior to C-19’s arrival, the pandemic perturbed operations significantly. Actions taken by AHS units that were aimed at rapidly deploying mass C-19 testing across the province initially did not include provision for test result notifications to be shared with PCPs.^14^ This emergent integration challenge^15^ was met with parties in the 2 siloes building a data bridge between C-19 clinics run by the PCNs and the AHS public health and testing units generating the results.^13^ The integration enabling data bridge solution ensured PCPs knew about C-19 positive patients, and that those patients received in-community follow-up. Staff from the PCNs- and AHS personnel also combined to develop an evidence-based clinical algorithm that supported PCPs in providing medical and social care to C-19 positive patients in the community. Emergency Departments in AHS hospital facilities were made aware of the follow up strategy, and knowing it was in place altered their threshold for admission allowed them to discharge “borderline” patients knowing they would be followed for deterioration.^14^ The pandemic, then, was a generative moment in the integration history of the primary and acute care elements of Alberta’s system. It provided the impetus for overcoming persistent barriers to integration, and, as we will show, the enactment of the province’s PHC goals through its specific commitment to the PMH.

### The Patient’s Medical Home

The broad goals of PHC, set out in the 1978 Alma-Ata declaration,^38^ can be described as providing widely accessible first-line care focused on patient needs and experiences that goes beyond, and wraps around, those patient’s bio-medical conditions.^39,40^ This is to say, PHC works with the social, as well as the bio-medical, determinants of health.^41,42^ It offers patients convenient, comprehensive, future-focused care delivered over time and in the context of a continuous relationship.^9,39^ It involves not just care delivery, but co-ordination, with an emphasis on *teams* of clinicians working together and at the top of their members’ licensures.^43-45^ A decade or more ago, these broad PHC concepts were distilled into the Patient Centered Medical Home care model in the United States.^46-50^ That model has shown significant promise in reducing costs and improving outcomes since.^49^ Renamed the PMH as part of its adaptation to Canadian primary care conditions,^25,51,52^ the model has subsequently been adopted across the country^53,54^ having been assessed, revised, and evaluated in terms of general policy preparation and uptake^54-56^ as well as for the factors shaping and shaped by the members of its teams.^57,58^ Table 1 highlights key elements of the PMH as deployed in Canada.

**Table 1.** Key Elements of the Patient’s Medical Home.

Patient’s medical home key elements		
Patient centered	Team-based medicine^a^	Continuous home
• Care is accessible by, and organized around, the patient’s bio-medical *and* social situation.	• Health and Allied Health professionals collaborate to work at the top of their licensure.	• The patient has access, over time, to team members whom they know.
• The team not only provides collaborative care internally, but coordinates it externally.	• Through coordination, the team is extended beyond primary care.	• The full team is familiar with each patient’s bio-medical and social history.
• Information Technology is used to engage proactively with individual patients inside panel populations.	• The team is extended to include data analytics and quality improvement personnel who work with population data to provide bespoke care.	• The team emphasizes prevention and wellness over the course of long-term relationship.

a A range of organizations and policies have moved away from the bio-medical centrism of this formulation, using “health home” interchangeably with “medical home.” We focus here on the PMH as it is official policy in Alberta^26,44^ and suggest that, semantic debate notwithstanding, the aspirations and methods of the Health Home are substantially similar to those of the Medical Home.

In March 2020, when Alberta’s first C19 case was identified, as with other PHC organizations,^11-14^ PCNs pivoted to managing patients in the community.^13,14^ They brought to this pandemic response work not just their resources, but their mandated commitment to the PMH.^26^ This is to say, they brought the PMH as an operational model to the work of making the principles of PHC real under pandemic conditions. In what follows, we highlight how C19 interacted with and informed PHC stakeholders’ approach to, and operationalization of, the PMH. We draw broader lessons for those seeking to continue the transformation of primary care toward PHC principles and the enactment of the PMH.

## Methods

The qualitative data presented in this paper are from a broader mixed-methods research study that sought to understand: (1) if, and how, patients of a PCN-based C19 clinic were already connected to a PMH; and (2) if, and how, any new patients were matched with a PCP in the process of becoming attached a PMH for the first time^13^ In the course of the analysis and coding process aimed at answering these questions our team identified crosscutting themes that highlighted the PMH and its underlying PHC principles being put into action under pandemic conditions. In this way we shifted from a deductive project aimed at answering a priori questions to a deductive emergent analysis driven by the collected data.

To gather the qualitative data presented here, we completed semi-structured interviews from January 2021 through to March 2021 with a range of stakeholders whom we identified using purposive (https://methods.sagepub.com/foundations/snowball-sampling) and snowball sampling techniques.^59,60^ The project’s Scientific Advisory Committee identified prospective participants and selected them based on their involvement and influence in shaping the primary care response to C19, ensuring that AHS, PCNs, and physicians were represented amongst those interviewed. An e-mail invitation was sent to 79 individuals identified by the Scientific Advisory Committee and as people consented to take part, interviews took place and recruitment continued. Of the 79 identified, 57 individuals agreed to participate and completed an interview that lasted between 19 and 71 min (average length = 35.7 min, median length = 32.5 min). PCNs employed 39 participants (eg, executive directors, physician clinical directors, clinic managers, physician leads, and technical analysts who supported the data sharing process), AHS employed 10 (primary care medical directors and executive directors and managers of AHS clinics that coordinated with PCN clinics and leadership), and community physicians connected to PCNs represented the final 8 participants (Table 2).

**Table 2.** Participants Gender and Role.

Study population	Numbers
Female	37
Male	20
Alberta Health Services—Public Health	1
Alberta Health Services—Primary Care • *Leadership, Administration, Data management*	5
Calgary Zone Primary Care Network • *Administration, Clinical Leadership, Data management*	38
Independent clinicians affiliated with PCNs	10
AHS-run clinics (C4 clinic) • *Administration, Clinicians, and Data management*	3

Interviews were conducted by 2 members of the research team with extensive experience in qualitative research. Almost all one-on-one interviews were conducted virtually (ie, using Zoom), with 2 of the 57 interviews including 2 or more participants. We emailed all participants an informed consent from prior to participation, along with a copy of the interview questionnaire so that they could consider the questions in advance. Interviews were audio-recorded and subsequently transcribed verbatim. In addition, we produced a summary cover sheet immediately following each interview to capture emerging observations and themes.

Interviews explored the integrated response between the central health authority (ie, AHS), the PCNs, and community physicians. Interviews aimed to understand how the people, organizations, and relationships facilitated or inhibited the C19 response initially, as well as how these changed throughout the course of the response during the first wave of C19 infections. Interviewers used open-ended, narrative interviewing, which is a method of qualitative data gathering that encourages participants to speak to their own experiences in whatever order they feel is most meaningful, allowing the participants to prioritize their perspective rather than adhering to a strict research agenda.^61^ Using a semi-structured interview guide, interviewers asked questions and identified probes; however, interviewers followed the participants’ lead and what the latter chose to prioritize and share during the interview based on their own experiences.

This study utilized the Interpretive Description approach. This analytical approach involves a continuous relationship between data collection and analysis, whereby interviews are conducted and field notes (interview summary cover sheets) are taken to capture observations made during the data-gathering process that can help contextualize the data during analysis.^62^ Upon completion of the interviews, the 2 researchers who conducted the interviews reviewed the transcripts and cover sheets to orient themselves to the data and develop a set of preliminary codes. They performed this first cycle of open coding until they assigned each segment of text a conceptual code that described its contents. Qualitative researchers employed the constant comparative approach, whereby they developed each code in consideration of all other codes in order to determine analytic distinctions.^63^ Once the data were coded, a third analyst completed a second focused phase of coding to identify patterns and develop themes that conceptualized larger segments of the data. Analysis was considered complete when the coding became saturated, and the established themes adequately described the patterns in the data. While we did not seek participant feedback on the analysis, we did review a high-level summary of emerging themes as a group (which included primary care physicians). This study was approved by the University’s Conjoint Health Research Ethics Board (REB20-0959).

## Findings

Key themes found in the data included: the importance of the PMH to overall PCN operations and the impact of C19 on those operations; and approaches to maintaining patient centric, team-delivered, and continuous care.

### Importance of PMH and Impact of C19

PCN staff and physicians emphasized the importance of the PMH to their organizations’ operations. They described how PMH tenets were foundational to normal operations, with a focus on ensuring access to well-coordinated care (Table 3, Quote 1). The pandemic, however, introduced a specific access issue that extended beyond the PCNs’ world: hospital capacity (T3, Q2). Protecting acute care’s capacity to deal with the sickest patients became a key organizing principle as the PCNs responded to the pandemic (T3, Q3). In these pandemic induced adaptations we see the PCN teams seeking to deliver on their specific PMH mandate, and so on the broader PHC principal of ensuring access, by expanding their definition to include the protection of central hospital capacity through the management of C19 patients in the community.

**Table 3.** Verbatim Quotes in Findings.

Quote no.	Partici-pant no.	Quote
1	031	“[As PCNs] we are trying so hard to advance [the] Medical Home framework . [I]n the simplest terms [that’s the care model] where every person gets a primary care provider that they have access to [and] continuity with [that provider]. [The patient and provider] develop that rapport and relationship [over time]. . . .Care coordination and team based care are central, [with the] PCNs essentially employ[ing] the staff to do the care coordination [work and be the] team. [We also] assist our members in knowing what the medical framework is and advancing the concepts of access and proactive care and all of that. [As PCNs] we’re always viewing what our role could be [through that lens].”
2	006	“I think the PCNs see themselves as advocates for the public in terms of [promoting and facilitating] health access. . .[And] in the pandemic, as the first wave hit, [we asked ourselves:] ‘What can we do to help contribute to this solution?’ [I think we] recognize[d] the opportunity to look at developing access points for the public that were easy [to find and navigate].”
3	008	“96% of the patients don’t need the hospital so we wanted to provide medical follow up to reduce unnecessary visits to Emergency Departments. [The idea was to] activate the 1,500 family physicians in the [Calgary Zone and so] reduce the burden on acute care. Because we knew [the first wave of the pandemic] would be won or lost in our hospitals.”
4	037	[Local hospitals set up what we called] a navigation team. These were [made up of] mostly nurses [and] there was one social worker. There would be one [team] in each of the major 4 urban hospitals. When [an in-patient] with complex needs was being discharged, [the navigation team] would [do] a warm hand off to the family physician saying ‘Look [this patient’s] been discharged, [they’ve] just been diagnosed with diabetes and had a heart attack. So we know [they need] follow-up care in the community just to make sure he isn’t re-admitted within 30 days.’”
5	037	“[Just before C19 we had] set up a pilot [program] with emergency departments (EDs) as well. [Like with the in-patient navigation team, the ED team] can connect into a specific PCN to make sure that when [their patient] leaves the ED they can either be followed up by their current [family] doctor, or if the patient [says]: ‘Yeah maybe I should get a family doctor because this abscess that I [came to the ED for] may last for several months. Maybe it’s time to get a family doctor,’ Then the ED team can say ‘We can help out with that!’ So what [COVID] did was it opened the door to creating these partnerships. Because now everyone is totally trying to prevent the inpatient system from being overwhelmed. . .So any partnership that will help with that, everyone is really open to it. I think that that will remain a success story. Because I don’t imagine why [the hospital in-patient or ED services] would revert back to saying ‘Well we don’t care if hospital beds fill up!’ [As a result of the pandemic] we’re starting to get headways into the [acute care] system where they realize ‘Oh when we partner with PCNs it actually reduces their risk of readmission or, at least we know the patient is being followed up after they leave.’”
6	020	“[Our PCN got involved] in doing some, for lack of a better word, ‘pop up swabbing.’ So we actually did some, some testing ourselves. [We saw our role as] to work with other agencies including AHS [and] ensure that we were all working together and were patient centric. [The broader aim was] where possible to keep the patient out of the hospital and keep them at home in their community with all the information and resources they needed to stay isolated when they were ill.”
7	015	There was an outbreak [in a ski resort town nearby]. Their little grocery store was closed. The bakery was closed. If you worked for a large employer you had food provided for you. Because [those big employers] have kitchens and your accommodation is part of that facility. But other [workers] were kind of high and dry. So here’s the beauty of it all: I got an email from [the mother of a patient] from Australia who said ‘My son has COVID, he needs medications and he needs food, obviously we’ll pay but can you help me?’ I was able to say ‘Yes of course’ and [I sent that request] to the manager at the [nearby PCN] clinic and [they were able to] hook this young man up in several hours.”
8	010	[According to] public health [regulations when you test positive for C19] you’re supposed to stay at home for 10 days; if you’re a contact [it’s]14 days. For people who are pretty well off, you know it’s not that burdensome. [But] for people who have jobs that don’t [include] sick leave – people who have jobs that don’t pay very much – [for those people] it’s a huge problem actually, right? This manifested itself with the whole [meat packing plant] thing where [they] had the largest single outbreak in North America: 1000 cases known amongst the workers themselves; 500-600 cases among family members. [These are] close to minimum wage workers. [They] don’t speak English as a first language. [They have] maybe limited health literacy. And [we] ask them to stay home for a long time. [But] if you need to feed your kids the only way you do that is by going to work. They gotta work right? [Under those circumstances] I think that providing [social] support is quite critical.
9	034	“I’ve been in practice for 21 years and I would say it’s only been over the last couple of years where, when I’m talking to my diabetic [patients that things have shifted. Even if my first thought still is] ‘Why are we not getting control over your diabetes?!’ I’ve been able to shift gears [and ask instead] ‘Are you having trouble affording food? Are you buying crappy food because it’s cheap and that’s the only thing you can afford and here I’ve been lecturing you all these years about eating fruits and vegetables. And in the back of your mind [you’ve been thinking], I can’t afford that!’ [That] conversation around the social determinants of health, and shifting care to the community, and keeping people out of hospital was already starting to happen pre COVID. [During the pandemic] I think the guidance we got from [AHS’s public health unit on] making sure we kept those social red flags in [mind] made doctors do some work that they probably should have been doing all along.”
10	024	“[During that first wave of COVID] we really wanted to ensure that patients were being taken care of holistically and that we were looking at all of those different social determinants of health. Providing [them with] resources and navigation [tools] for things like food security, mental health, [even] questions about [the] isolation hotels [that were set up by the province]. We tried really hard to not just follow [a bio-medical care pathway] and carry on. We really wanted to make sure that patients were taken care of in that holistic way that we would ordinarily take care of [them].”
11	047	“[In the first wave of the pandemic] we opened up what we call a COVID Access Clinic. At that time . . .the evidence was just turning over so quickly [that] people didn’t really know what we were dealing with. We had a number of physician [members] that were, I mean, I’ll use the word ‘scared.’ I don’t know if that’s the right word but [they were] unclear and, and just a little bit uneasy. [They were asking themselves:] “Well should I see these people, or should I not?” So what we opened our clinic initially for was to support the physician [members with these doubts] to be able to actually see COVID positive patients.”
12	016	“At the beginning we relied heavily on [the central system and the] information that they could provide us with. . .And then the collaboration with the other PCNs in Calgary [began and we started] trying to come up with processes and protocols for how we were going to treat [COVID positive] patients. [Some family physician members] were really quite leery [of seeing patients] because they didn’t have any information, so [the PCNs] probably did 99% of following patients through our Access Clinics and we were bumping up staffing and physicians to manage the patient care.”
13	038	“Basically, Access Clinic [work] is shift work. You pick up shifts during the day or the evening or the weekends. It’s a sign up process [that draws volunteers from] member physicians. You have to be a member [of the relevant PCN to work at] that Access Clinic. Usually it would average 1 shift a week, maximum 2 shifts a week. It’s like a 3 hour shift and you’re seeing patients . . . who are referred to you through [the central system] or if [their] family doctor is. . .too full, or they can’t see them, or they’re on holiday. . . As soon as you finish seeing [the patient, their regular family doctor] gets sent a fax summary of the encounter.
14	025	“[Our PCN has] a 24 hour blood pressure monitoring program that we do normally. Monitoring is available elsewhere in the community but we do it with no charge, and we have a shorter wait time for our physician members to refer their patients into [our program]. That was deemed something that could be put on hold until we had the capacity to resume. We redeployed staff [from there] at various times to the Access Clinics.”
15	004	“Our [PCNs’] Allied Health Team – so our pharmacist, dieticians, physio and occupational therapists – they were also involved [in the Access Clinics] because a lot of their disciplines were shut down with the first [wave of the pandemic]. . . We needed the bodies!. . . [With them] we had the manpower to go through and do a lot of the administrative functions of the Access Clinic program.”
16	031	“[A lot of the Access Clinic shift work] was so complex, right? You’re looking at [the patient’s] social needs, [their] food security, their living arrangements] for isolation purposes. Doctors were spending about an hour on the phone assessing everything. So we [began redirecting] all the socio economic assessment [to PCN] nurses.”

Over the course of the pandemic, to ensure access to PHC, PCNs expanded their capacity to coordinate care with centrally managed acute care facilities. PCN staff integrated into navigation teams that were set up by local hospitals to facilitate the discharge of post-C19 in-patients who also had complex chronic conditions. These warm hand-offs from acute- to primary-care (T3, Q4) extended the care delivery team beyond PHC, increased coordination and mutual trust between the 2 parts of the health system, reduced hospital readmissions, and sought to attach patients to a PMH if they did not already have 1 (T3, Q5). Similarly, in the Emergency Department setting, a pre-pandemic and PHC-focused partnership between hospitals and PCNs aimed at coordinating care in the community and attaching patients to a PMH gained momentum as C19 struck (T3, Q5).

### Approaches to Maintaining Patient Centric, Team-Delivered, and Continuous Care

PCNs serving rural areas sought to ensure access by opening their own C19 testing centers. In Alberta, testing was a central system mandate, but the drop-in and drive-through facilities were often distant from rural patients’ homes. In response, a rural PCN adhering to the PMH’s patient centric principals, opened its own “pop up swabbing” center (T3, Q6). With rural Alberta hosting large populations of both short-term international workers and permanent immigrant and refugee newcomer workers, local PCNs encountered particular access-to-care issues in these lower-income populations. Short-term international workers in locked-down ski resorts found themselves without access to food and medication. PHC teams of physicians and PCN staff following the PMH’s holistic and relationship-focused principles responded to this combination of food insecurity and medical need (T3, Q7). In an adjacent PCN’s catchment, lower wage immigrant workers at a rural meat packing plant were similarly prioritized for access to team-delivered holistic social and medical supports (T3, Q8). While the patient centric foci of ensuring access and addressing the social determinants of health were not new, the pandemic sharpened family physicians’ focus on providing care that went beyond bio-medical considerations (T3, Q9-10).

In urban areas, efforts to ensure patients had convenient access to PHC focused on the creation of C19 Access Clinics. These clinics emerged in the first wave of the pandemic as individual family physician members of the PCNs wrestled with fears and incomplete or varying information on the virus and the number of appointments available to patients dropped (T3, Q11). Relying heavily on information supplied by central AHS systems, staff from the various urban PCNs began collaborating to expand and adjust the way the C19 Access Clinics worked and to safeguard patient access to PHC (T3, Q12). Family physicians worked shifts at the clinics, providing access to care in the absence of in-PCN colleagues (T3, Q13). For their part, the PCNs themselves redeployed allied health staff from other PMH-focused programming that had been suspended due to the pandemic (T3, Q14-5), reconstituting and expanding the team that was delivering care. These redeployments backstopped family physicians who were working shifts at the Access Clinics and spending far longer to understand and respond to the social determinants of their patients’ health than was covered by the available FFS billing codes (T3, Q16).

Our data highlights, on the one hand, that primary care teams protected the acute care system’s capacity. On the other, we have shown that those same teams ensured access to PHC in the community. Protecting hospital capacity became a shared mission that transcended the Alberta health system’s silos of “independent FFS primary care” and “centrally funded acute care.” If this became a shared mission, the words of the shared narrative that supported it were taken directly from PHC principles and provincial PMH policies. The PMH care model became the words and ways to think about, engage with, and respond to the C19 shock.

## Discussion

As shown, PCN staff and family physicians, as well as their counterparts in hospital in-patient and Emergency Departments, saw their pandemic mission as being one of ensuring access. Access has been defined broadly as “the degree of fit between clients and the system”^64^ with 5 specific dimensions of this “fit” identified as: availability, accessibility, accommodation, affordability, and acceptability,^58^ with a sixth dimension “awareness,” added more recently.^61^ Our participants’ efforts to protect the acute care system by diverting patients or managing them in-community to prevent re-admission were focused on “availability.” Which is to say, the mission and narrative shared by primary care and acute care personnel hinged on balancing the relationship between the volume of existing services and resources and the volume and type of patient needs.^65^

While this dimension of access was clearly important in the thinking and actions of independent primary care stakeholders as they built the C19 Access Clinics, our data suggest the pandemic encouraged gains in other aspects of access as well. Specifically, “accessibility,” “accommodation,” and “awareness” were particularly targeted by primary care teams as they used the principles of PHC and policies of the PMH to make sense of and respond to the pandemic. As evidenced by the efforts rural locations to open pop-up testing centers, *accessibility* in access was sought through efforts to optimize the relationship between the location of primary care supply and patients’ locations. As evidenced by the efforts to address food insecurity as well as biomedical issues, *accommodation* in access was sought through efforts to tailor care availability to the temporal, spatial, and social needs and capacities of patients As evidenced by efforts to integrate PCN staff into acute-care-based navigation teams, *awareness* in access was sought through efforts to optimize the flow of well-adapted communications and information.

Grounding these efforts to ensure access—arguably the defining feature of PHC^66^—was the emphases and practices of the PMH. *Patient centrism* was expressed as a focus on not just bio-medical conditions, but the social determinants of health. It saw primary care teams create C19 Access Clinics, pop-up swabbing stations, food security interventions, isolation management solutions, and it saw them altering their clinical encounters, all so that patients were met where they were spatially, bio-medically, and socio-economically. *Team-based medicine* was made a practical reality in partnership and care-coordination efforts that extended well beyond the primary care clinic, and in the redeployment of PCN staff to distribute the work required to deliver patient centrism under pandemic conditions. Establishing a *continuous home* connection with more patients—indeed “all Albertans” as called for in policy^26^—was pursued through partnerships with acute care stakeholders who, as C19 shocked their capacity, came to understand the value of working with primary care and so of attaching patients to a medical home.

In these examples of the pandemic pushing progress toward PHC, there are also unanswered questions. Our methods, as with all qualitative studies, offer a cross-sectional study of a moment in time and, notwithstanding our participants’ hopes that relationships and improvements to access would survive the waning of C19, it is unclear whether the gains we describe here can be maintained. Given that the pandemic rendered the redeployment of a small army of PCN staff possible, how might the accessible patient-centric care that was delivered under such heightened circumstances be supported in less fraught times? As jurisdictions around the world face strained primary care systems and demands for reform or transformation grow,^67-69^ more research is required to understand how not just to embed the narrative and mission of PHC in stakeholders’ minds and practices, but to rebalance funding and incentives to support the PMH beyond the C19 shock.

## Conclusion

More than 45 years after the Alma-Ata Declaration, the C19 pandemic delivered a shock to primary care systems around the world. In the intervening time, the Declaration’s PHC principles had become the basis of widely adopted care models like the PMH. In the case of Alberta’s PCNs, the PMH provided the words, and ways, to think about, engage with, and respond to the C19 shock. It focused primary care personnel on the various dimensions of care access, and through its emphasis on patient centrism, team-based delivery, and continuity of care, saw them flexibly adapting to changing conditions. Indeed, the pandemic provided an opportunity for operationalization of PHC principles, and created a foundation of new relationships and ways of working to further explore PHC tenets. The extent to which these gains are shared in other jurisdictions, and the PMH narrative can be embedded in normal non-pandemic operations as health system reforms are undertaken, remain open questions.

## Supplemental Material

sj-docx-1-jpc-10.1177_21501319241236007 – Supplemental material for Thinking and Enacting the Patient Medical Home Under Pandemic Conditions: A Qualitative Study From Primary Care in Alberta, Canada Supplemental material, sj-docx-1-jpc-10.1177_21501319241236007 for Thinking and Enacting the Patient Medical Home Under Pandemic Conditions: A Qualitative Study From Primary Care in Alberta, Canada by Myles Leslie, Brian Hansen, Rida Abboud, Caroline Claussen and Fariba Aghajafari in Journal of Primary Care & Community Health
