# Supplementary material for: Thinking and Enacting the Patient Medical Home Under Pandemic Conditions: A Qualitative Study From Primary Care in Alberta, Canada
Source: J Prim Care Community Health. 2024 Apr 16;15:21501319241236007. doi: 10.1177/21501319241236007 (PMC11022528; doi:10.1177/21501319241236007)
Supplement: sj-docx-1-jpc-10.1177_21501319241236007 – Supplemental material for Thinking and Enacting the Patient Medical Home Under Pandemic Conditions: A Qualitative Study From Primary Care in Alberta, Canada [file sj-docx-1-jpc-10.1177_21501319241236007.docx]

Appendix 1. Interview Guide

This is a study about a pair of interventions that have been labelled the “COVID-19 Integrated Pathway” (the CIP). Those interventions are: 1) a ***data pathway*** between provincial lab services, public health, AHS primary care, and PCN primary care, and 2) a ***clinical algorithm*** created to support family physicians in delivering care to SARS-COV-2+ patients. You might know this second one as: ***The COVID-19 Clinical Pathway.*** We want to understand the people, organizations, and relationships that made both of these elements of the CIP come together initially; change over time; and potentially spread across the province.

Q1: Is the CIP in one, or both, of these interventional forms, a label or activity that you’ve come across since the pandemic started?

Q1FU1: If yes, when and in what capacity? If no, end interview.

Q1FU2: Were you part of designing, delivering, or updating either or both parts of the CIP?

Q1FU3: How so, what was your role specifically?

Q1FU4: How did you come to have that role?

Q1FU5: Did that role change over time?

Q2: How, if at all, have the two interventions in the CIP–the ***data pathway***, and the ***clinical algorithm*** – shaped your work during, or experience of, the pandemic?

Q2FU1: Do you see the two interventions as related?

Probe: We have begun to see the clinical algorithm as the end point of the data pathway, but are not sure how much uptake of the clinical algorithm there is amongst clinicians.

Q2FU2: Do you see places where one or both of the interventions could be made more effective?

Focusing in on the ***data pathway***, I’d like to talk about how you saw it evolve over the course of the pandemic.

Q3: What, in your mind, made it necessary/desirable in the first place?

Q3FU1: What has worked well with the data pathway?

Q3FU2: What challenges have there been?

Probe: technology, policies, communication, resources

Q4: Were there particular people or organizations that made that first iteration possible?

Q4FU1: Who were they?

Q4FU2: How did they share their ideas?

Q4FU3: How did they gain consensus/buy in/resources for the idea?

Q4FU4: Are any of those people, organizations, meetings, or consensus techniques critical to the export of the idea to another zone?

Q4FU4FU1: If yes, which ones and how so?

Q4FU4FU2: Can you think of ways that the idea might be exported or scaled without some or all of those people, organizations, meetings, or consensus techniques?

Q5: How did you, or others, determine that the pathway process needed to be adjusted/evolved (i.e., what were the signals?)

Q5FU1: What resources or relationships made those adjustments/evolutions possible?

Q6: Were there particular people or organizations that made the evolutions and iterations possible?

Q6FU1: Who were they?

Q6FU2: How did they share their ideas?

Q6FU3: How did they gain consensus/buy in/resources for making the changes?

Q6FU4: Are any of those people, organizations, meetings, or consensus techniques that are, in your mind, critical to the export of the idea to another zone?

Q6FU4FU1: If yes, which ones and how so?

Q6FU4FU2: Can you think of ways that the idea might be exported or scaled without some or all of those people, organizations, meetings, or consensus techniques?

Q7: What, in your experience, was the most important or salient element of the ***data pathway*** of the CIP?

**Prompt** (data capture, data management, patient attachment, patient contact)

Q7FU1: How so?

Q7FU2: Were there particular benefits or drawbacks to this data pathway?

Focusing in on the ***clinical algorithm – which you may know as the COVID-19 Clinical Pathway*** - I’d like to talk about how you saw it evolve over the course of the pandemic

Q8: Did you and/or your colleagues make use of the ***clinical algorithm***?

Q8FU1: **If yes**, How does the algorithm fit into your clinical management practices for COVID-19 positive patients?

Are there benefits and/or drawbacks?

Have you developed new protocols or workaround in your practice, or EMR to deal with these?

How have you handled the scheduling and delivery of follow up calls, particularly for high risk patients who may need follow up in off hours?

How is the algorithm facilitating, or hindering the transfer of care responsibility from AHS-PH/C4/PCN Access into your clinic?

What, if anything, could be improved?

How do you find patients are reacting to the follow ups or other algorithm-induced interventions you are making?

Q8FU2: **If no**, or limited use, What were the factors that drove your decision not to use the algorithm?

Q9: Do you see the ***clinical algorithm*** as integrating with the ***data pathway***?

If the CIP – both the clinical algorithm and the data pathway – were an attempt to integrate Provincial lab services, AHS Public Health, AHS Primary Care, and the PCNS,

Q10: Was the attempt at integration successful?

Q10FU1: If yes, what would be required to move this integrative approach outside the Calgary Zone?

Q10FU2: If no, are there any factors that should be considered in improving the CIP’s capacity to integrate these parts of the health system?

If NO: What tools are you using to manage your patients (Prompt – inside your EMR? Via college or AMA? Nothing at all? How do you manage

Q11: Any thing else? Who else should we be interviewing on this topic?
